# Analysis of Walking Economy after Sleeve Gastrectomy in Patients with Severe Obesity

**DOI:** 10.3390/biology12050746

**Published:** 2023-05-19

**Authors:** Marco Vecchiato, Sara Faggian, Giulia Quinto, Francesca Battista, Mirto Foletto, Angelo Di Vincenzo, Silvia Bettini, Andrea Gasperetti, Luca Busetto, Andrea Ermolao, Daniel Neunhaeuserer

**Affiliations:** 1 Sports and Exercise Medicine Division, Department of Medicine, University Hospital of Padova, Via Giustiniani 2, 35128 Padova, Italy; marcovecchiato.md@gmail.com (M.V.);; 2 Clinical Network of Sports and Exercise Medicine of the Veneto Region, 35131 Padova, Italy; 3 Bariatric Surgery Unit, University Hospital of Padova, 35131 Padova, Italy; 4 Center for the Study and Integrated Treatment of Obesity (CeSTIO), Internal Medicine 3, Department of Medicine, University Hospital of Padova, 35128 Padova, Italy

**Keywords:** obesity, BMI, energy expenditure, walking cost, bariatric surgery, cardiopulmonary exercise testing

## Abstract

**Simple Summary:**

Patients with obesity have a higher energy cost of walking than normal-weight subjects. Can bariatric surgery help these patients to improve their walking economy? In this study, a group of patients underwent cardiopulmonary exercise testing one month before and six months after laparoscopic sleeve gastrectomy and their parameters were registered at three different submaximal protocol stages. As well as significant weight loss, improvements in walking economy were evident as early as six months after bariatric surgery even when patients were grouped by gender and obesity class. These changes result in less impairment when performing daily physical activities.

**Abstract:**

Background: Obesity is associated with a higher energy cost of walking which affects activities of daily living. Bariatric surgery with sleeve gastrectomy (SG) has beneficial effects on weight loss and comorbidities. Purpose: The aim of this study was to analyze the impact of SG on walking economy in subjects with severe obesity. Methods: This observational cohort study included all patients with morbid obesity who were considered suitable candidates for SG between June 2017 and June 2019. Each patient underwent an incremental cardiopulmonary exercise test on a treadmill (modified Bruce protocol) one month before and six months after SG. Data on the energy cost of walking were recorded during three protocol stages (stage 0—slow flat walking: speed 2.7 km/h, slope 0%; stage ½—slow uphill walking: speed 2.7 km/h, slope 5%; stage 1—fast uphill walking: speed 4.0 km/h, slope 8%). Results: 139 patients with morbid obesity (78% women; age 44.1 ± 10.7 years; BMI 42.5 ± 4.7 kg/m^2^) were included in the study. At six months post-SG, patients presented with a significantly decreased body weight (−30.5 ± 17.2 kg; *p* < 0.05), leading to an average BMI of 31.6 ± 4.2 kg/m^2^. The net energy cost of walking (measured in J/m and J/kg/m) of the subjects was lower compared to pre-SG at all three protocol stages. This improvement was also confirmed when the subjects were grouped by gender and obesity classes. Conclusion: After a significant weight loss induced by SG, regardless of the severity of obesity and gender, patients exhibited a lower energy expenditure and an improved walking economy. These changes make it easier to perform daily routines and may facilitate an increase in physical activity.

## 1. Introduction

Obesity is a multifactorial disease, defined as an abnormal or excessive accumulation of fat, and is diagnosed at a body mass index (BMI) over 30 kg/m^2^ [1]. Living with obesity increases the risk of disability, several diseases, and comorbidities, including diabetes mellitus, dyslipidemia, cardiovascular diseases, and cancer [2,3,4]. The prevalence is high, increasing in dimension over the past 50 years [5], while it currently continues to grow [1,6,7] even during the last global pandemic [8,9]. Multicomponent behavioral interventions are generally considered to reduce calorie intake and increase energy expenditure [5]. However, when healthcare systems fail to treat obesity in its early stage with non-surgical interventions (i.e., lifestyle modifications and pharmacological treatment), severe obesity occurs, leading to the point that bariatric surgery is needed [5]. Sleeve gastrectomy (SG) is an important treatment option for morbid obesity that results in sustainable weight loss and has positive effects on various metabolic and cardiovascular conditions [10]. A mean weight loss three years after surgery of 16% made SG a feasible and successful bariatric surgery intervention [10,11]. However, to achieve less weight regain, maintenance, or even greater weight loss over time after surgery, increasing physical activity (PA) and reducing sedentary behavior play a key role [12,13,14].

PA accounts for 15–30% of total daily energy expenditure and includes two components: purposeful-voluntary exercise activity and non-exercise activity thermogenesis (NEAT), e.g., standing, stair climbing, pacing on the phone, cleaning the house, walking the dog, which can vary for a given person by as much as 2000 kcal per day [15,16]. Indeed, obesity is associated with low NEAT; people with obesity stand and walk for 2.5 h per day less than their lean sedentary counterparts [16,17]. In line with this, a low exercise economy has been found to be significantly related to reduced NEAT [18]. Since walking is the predominant component of PA [17,19], it is noteworthy that patients with obesity demonstrate a higher energy cost of walking compared to normal-weight individuals [20,21,22,23], which may limit regular engagement in NEAT [24,25]. Moreover, some studies have recently shown that obesity classes are associated with walking efficiency [21]. The higher energy cost may contribute to fatigue, physical inactivity, and increased sedentary time, thus reducing the quality of life and the effectiveness of exercise programs in weight management [26,27,28]. Therefore, it is important for patients with obesity to select activities that are pleasing and easy to perform [16], considering the simplicity of locomotion as a critical component for long-term adherence to PA and exercise [26].

Weight loss following non-surgical interventions and the associated increase in relative maximal oxygen consumption contribute to an improved walking economy in overweight patients and patients with obesity [29,30]. Some pilot projects have investigated the effects of bariatric surgery on oxygen consumption, energetics, and the mechanics of walking in small samples of patients with obesity [31,32]. However, the impact of SG on such submaximal functional performance data has not yet been investigated in a large sample of patients with severe obesity by performing the gold standard evaluation of cardiorespiratory fitness, i.e., cardiopulmonary exercise testing (CPET). Therefore, the purpose of this study was to determine the effect of a very large body mass loss induced by SG on functional capacity through walking economy analyses in a large study population of patients with severe obesity.

## 2. Materials and Methods

### 2.1. Participants

All patients affected by moderate–severe obesity who were suitable candidates for SG after a multi-disciplinary evaluation were consecutively recruited for this observational cohort study. The study was conducted by the Sports and Exercise Medicine Division of the University Hospital of Padova between June 2017 and June 2019. All study participants followed a clinically approved pathway and underwent SG according to the Veneto Region resolution n.55/ CR 4 August 2015. The study was conducted in accordance with the Declaration of Helsinki and approved by the local Ethical Committee for Clinical Research (protocol code 2892P). Written informed consent was obtained from the participants before conducting a complete functional evaluation, which included anthropometric data assessment, medical examination, and CPET one month before and six months after SG. Participants who rejected surgical intervention, those under 18 years of age, and subjects with a history of psychotropic substance abuse or with cardiovascular/orthopedic diseases that contraindicated or impaired exercise testing were excluded from the study.

### 2.2. Sleeve Gastrectomy

According to international recommendations [33], bariatric surgery was performed in patients with a BMI higher than 35 kg/m^2^ in the presence of comorbidities or a BMI higher than 40 kg/m^2^. Absolute exclusion criteria included alcohol addiction and severe psychiatric disorders. Pre-operative evaluations included abdominal ultrasound, upper gastrointestinal endoscopy, and upper gastrointestinal barium X-ray. SG was performed as the first-choice procedure in our center, and all patients were operated on by the same bariatric surgery team. The surgical technique involves a stomach longitudinal resection, starting 4–5 cm from the pylorus with the preservation of the gastric antrum [34]. All patients received a very low-calorie diet (about 800 kcal/die) one month before surgery. After SG, all patients underwent regular scheduled post-bariatric follow-ups (after one month, six months, and twelve months) to monitor possible post-surgical complications and prevent nutritional deficiencies. In the early post-surgical period, a liquid diet was proposed, with the introduction of semi-liquid foods around the first-month post-SG. Multivitamin, protein, and mineral supplementations were prescribed to all patients.

### 2.3. Anthropometric Measurements 

Body weight was measured in underwear to the nearest 0.1 kg. Height was measured with a stadiometer to the nearest 0.5 cm. BMI was calculated accordingly to these measurements. Waist circumference was measured at the end of a normal expiration in the horizontal plane, midway between the superior iliac crest and the lower margin of the last rib, using a measuring tape placed horizontally around the abdomen and without compressing the skin.

### 2.4. Cardiopulmonary Exercise Testing

As previously described, each patient underwent a 12-lead ECG-monitored CPET (Jaeger Masterscreen-CPX, Carefusion) [35]. Careful calibration of the flow sensor and the gas analyzers was performed before every single evaluation according to the manufacturer’s specifications [36]. After an initial constant-speed test phase, the subsequent incremental part of the exercise test was performed until exhaustion. Criteria of exhaustion were a Borg rating of perceived exertion ≥ 18/20 associated with a respiratory exchange ratio (RER) ≥ 1.10, and/or a peak heart rate (HR) ≥ 85% of predicted maximal HR, and/or the achievement of a plateau of maximal oxygen uptake (VO_2_) [37]. METs max was determined by extracting the values of speed and slope obtained at peak exercise [38]. Respiratory gas exchange (VO_2_, VCO_2_) and ventilation were monitored breath by breath during the whole test and at least until the fourth minute of recovery. The VO_2_ peak was defined as the highest value of VO_2_ attained in a 30 s interval at peak exercise. The minute ventilation/carbon dioxide production (VE/VCO_2_) slope was calculated as the coefficient of linear regression obtained by plotting the VE and VCO_2_ data from the beginning of the exercise (removing possible initial hyperventilation) to the respiratory compensation point [39].

### 2.5. Protocol Stages and Energy Cost

**Figure 1:**
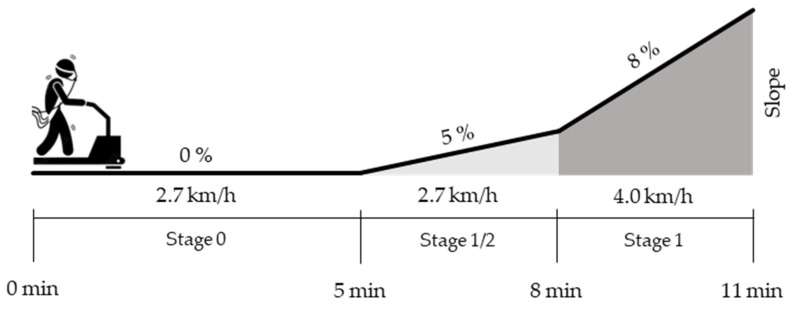
Protocol stages. Stage 0—slow flat walking: duration 5 min, 0% gradient, speed 2.7 km/h. Stage ½—slow uphill walking: duration 3 min, 5% gradient, speed 2.7 km/h. Stage 1—fast uphill walking: duration 3 min, 8% gradient, speed 4.0 km/h.

Tests were performed on a treadmill (T170 DE, Cosmed), according to a specific modified Bruce protocol, with an integrated initial 5 min constant speed interval (stage 0) to ensure familiarization with the test. The parameters registered at the various stages of the protocol were analyzed as the average of the values extrapolated in the last minute of the constant stage 0 (duration 5 min, 0% gradient, speed 2.7 km/h), of stage ½ (duration 3 min, 5% gradient, speed 2.7 km/h), and of stage 1 (duration 3 min, 8% gradient, speed 4.0 km/h) (Figure 1). The subsequent incremental stages were not included in the study because they were not reached by most patients and because they were affected by alterations in the walking mechanics (difficulty in maintaining a linear trajectory and holding the bar tightly). Due to a matter of safety, given the risk of falling resulting from an incremental protocol in this population, all tests were performed with patients holding the treadmill bar.

The gross absolute and relative energy cost of walking (J/m and J/kg/m, respectively) were calculated using VO_2_ values measured during the above-mentioned stages and also considering exercise intensity (i.e., RER), according to the formula of Garby and Astrup [40]:

Energy Cost = (4.94 RER + 16.04) × VO_2_/60

Subsequently, the net absolute and relative energy costs were calculated by subtracting the resting energy cost from the gross energy cost during walking [41].

### 2.6. Statistical Analysis

The Statistical Package for Social Science (SPSS Inc., Version 26, IBM Corporation, Armonk, NY, USA) was used for statistical analyses. The normality of all parameters was assessed using the Shapiro–Wilk test. Continuous variables were presented as mean ± standard deviation, and comparisons between the same population before and after SG were made using the paired samples *t*-test for normally distributed variables and related-samples Wilcoxon signed rank test for the non-normally distributed variables. All reported probability values are two-tailed, and statistical significance was considered at a value of *p* < 0.05.

## 3. Results

Out of the 139 subjects included in the study, 109 were women (36 with class II obesity and 73 with class III obesity), and 30 were men (10 with class II obesity and 20 with class III obesity). At baseline, 32 patients (23%) were affected by type 2 diabetes mellitus and medically treated with oral hypoglycemics, and 43 patients (31%) were treated for arterial hypertension. The general pre- and post-SG anthropometric characteristics of the study participants are represented in Table 1. Six months post-SG, patients lost an average of 30.5 kg (from 117.9 ± 19.6 kg to 87.4 ± 15.8 kg, 25.90 ± 5.34 % of body weight), leading from a mean BMI of 42.5 ± 4.7 kg/m^2^ to a mean BMI of 31.6 ± 4.2 kg/m^2^; (all *p* < 0.001). The mean resting systolic blood pressure before SG was 127.89 ± 13.32 mmHg, while at 6 months post-SG, it was 115.47 ± 4.66 mmHg; resting diastolic blood pressure decreased from 78.74 ± 10.32 mmHg to 72.83 ± 11.56 mmHg (both *p* < 0.001).

Furthermore, systolic blood pressure at peak exercise decreased from 179.09 ± 24.03 mmHg to 166.81 ± 25.79 mmHg (*p* < 0.001). All patients reached the criteria of exhaustion during the CPET. The weight modifications were also associated with an increased maximal exercise capacity and tolerance post-SG. Moreover, patients showed an improvement in exercise capacity and VO_2_ peak/kg, as well as a higher RER at peak exercise (all *p* < 0.001). However, after SG, a statistically significant decrease in absolute VO_2_ peak during exercise was shown (*p* < 0.001; Table 2).

Regarding walking economy at submaximal exercise intensities of different test stages, data showed lower HR and VO_2_ at similar metabolic exercise intensity (i.e., RER). Furthermore, all three protocol stages showed a reduction in the energy cost of walking for both absolute energy cost (J/m) and relative cost per kg body weight (J/kg/m) (Table 3). The same energy cost data were also examined by grouping subjects into gender and obesity classes. The differences for the overall sample were also confirmed for all subgroups analyzed.

## 4. Discussion

The main finding of the present study was an improvement in walking economy following SG, with a reduction in the absolute and relative energy cost of walking at different speeds and gradients.

SG has been proven to be highly effective in reducing patients’ weight and improving their cardiovascular risk and metabolic conditions [10,11,42]. Our results confirmed significant improvements with a mean decrease of 30.5 kg, approximately 26% of the initial weight and BMI, at six months after SG [42,43]. Specifically, both men and women recruited in our study went from moderate–severe obesity to overweight/mildly obese.

A significant improvement in cardiorespiratory fitness and work capacity at peak exercise has been confirmed in our study. Indeed, the maximum exercise and aerobic capacity of the study participants increased by 2 METs and 18.7% of VO_2_ peak predicted (corresponding to 4.0 mL/kg/min) until the second CPET evaluation, suggesting a better ability to perform the typical activities of daily life after SG [44]. As previously described by our group, absolute VO_2_ max decreased by 12.6% after SG (2.46 ± 0.58 L/min vs. 2.15 ± 0.55 L/min), likely due to the involvement of lean mass in weight loss [43]. Although accurate considerations regarding fat distribution cannot be drawn due to the lack of body composition data, the marked weight loss induced by bariatric surgery is probably not only associated with a reduction in fat but also, albeit to a lesser extent, in muscle mass, particularly in the first period after bariatric surgery [45,46]. Consequently, it could be assumed that the amount of muscle mass, which determines the total oxygen consumption, is reduced after the intervention, leading to a reduction in absolute maximum aerobic capacity [43].

It is known that patients with obesity show a marked impairment of functional capacity, leading to limitations in the ability to perform simple activities such as walking, getting up from a chair, and climbing stairs [47]. In addition, individuals with severe obesity show severely impaired cardiorespiratory fitness and exercise tolerance associated with a poor quality of life as they are easily fatigued and dyspneic during normal daily activities [48]. The aerobic capacity seems to have a well-defined relationship with walking economy. A work by Borges et al. on a sample of overweight women randomly assigned to different lifestyle and training interventions for weight reduction showed a good relationship between VO_2_ max and walking economy, persisting even after significant weight loss [29]. Thus, it seems that a better maximum aerobic capacity is generally associated with a lower VO_2_ for the same amount of required workload. On the other hand, few studies on small samples reported no difference between overweight and normal-weight subjects regarding gross energy cost per walking distance, attributing this equivalence to the lower resting energy expenditure of subjects with obesity, compensating for the higher net energy cost of walking [24,25]. Confirming what has been reported for non-surgical weight loss [29,30], in the present study, the net energy cost of walking obtained through the VO_2_ measurement at the three different walking speeds and gradients was significantly reduced post-SG, both when the net energy cost of walking is expressed in absolute terms (J/m), but interestingly also when expressed in relation to body weight (J/kg/m). However, in a recent work by Malatesta et al., no significant decrease in the relative net energy cost of walking per kg of body mass between pre and six months post-bariatric surgery was found, even though the body mass loss after surgery was quite similar to our work. Malatesta et al. suggested that loss of body mass, not fat mass, is the main factor involved in the improvement of walking economy in individuals with obesity after bariatric surgery [31]. However, since they found a significant difference in net energy costs of walking only 1 year after surgery, they hypothesized that factors other than body mass might have an influence on the energetics of walking, such as mechanical gait modifications. Additionally, the PA level was not considered as an influencing factor in improved walking economy since they did not register a significant change during follow-up. However, it seems reasonable that the changes in net relative energy costs of walking found in our study, even 6 months after surgery, could be influenced by the PA recommendations that patients received after functional evaluations before bariatric surgery, which possibly led to early mass-driven adaptations of gait behavior. When comparing the outcomes of both studies, the difference in sample size may also play a role (9 vs. 139 included subjects) [31].

Interestingly, in our study, the absolute and relative net energy costs of walking obtained through the VO_2_ measurement were found to be reduced after SG for all three intensity stages, even when the sample was classified by gender and BMI. It is known that both obesity and gender affect the net relative cost of walking. However, regardless of the severity of obesity, both men and women following major weight loss induced by SG faced the same workload with significantly less energy expenditure [49]. Although it has not been specifically addressed by our study, some pathophysiological mechanisms have been proposed for the underlying improvements in walking economy following major surgically induced weight loss. Moreover, Serés et al. observed a lower energy cost of walking at different intensities in 31 subjects one year after bariatric surgery [32]. However, given the lack of variation in peak oxygen pulse in relation to lean mass, they excluded the possibility that the walking economy improvements were due to better cardiac response to exercise, attributing it to increased exercise capacity [32]. Our study confirms the influence of cardiorespiratory fitness on the functional capacity to perform submaximal tasks with lower energy expenditure (~51% of relative VO_2_ peak at stage 0 before surgery vs. ~38% of relative VO_2_ peak at stage 0 after surgery). Furthermore, the RER peak suggests improved exercise tolerance after surgery, especially in those patients with class III obesity, while lower HR values were observed at submaximal intensities, indicating reduced cardiovascular efforts rather than optimization of the cardiac reserve, as described by Seres et al. [32]. Other works showed great improvements in walking economy following marked weight loss after bariatric surgery by ascribing this adaptation to a lower energy demand needed for movements and improved core stability [44]. Indeed, it is known that some biomechanical factors, such as reduced stability, a wider supporting base, and excessive lateral leg swing, contribute to a higher energy cost of walking for patients with obesity [20,50]. For example, in adolescents with obesity following non-surgical weight loss, the improvement in walking efficiency seems to be related to modifications associated with decreased metabolic rate to support the lower body and maintain balance [51]. According to previous studies, some biomechanical changes as the significant reduction in peak knee abduction/flexion and hip extension, are even more relevant than parameters of cardiorespiratory fitness and economy in improving the performance of daily activities as NEAT [52].

Regular PA and exercise training are beneficial for patients with obesity as they help to control risk factors and treat comorbidities [13,14,53]. The current literature suggests that, as early as three months after bariatric surgery, it may be useful to include an exercise program aimed at muscle strengthening, especially to help maintain lean mass [54]. Our unit provides exercise prescriptions to patients in different clinical conditions, including subjects with severe obesity before and after SG [55]. Since regular PA and exercise training are recommended before and after SG, it cannot be excluded that the improvement shown in the walking economy six months after SG has also partially been influenced by the PA performed.

### Limitations and Perspectives

This retrospective study investigated the walking economy using indices related to the energy cost of walking among CPET evaluations made for clinical purposes. For this reason, the stages of the protocol were not randomized between patients, and the walking economy was not assessed for a specified control group of normal-weight subjects. The major limitations of this study are the absence of gait analysis and body composition assessments; the discussion is thus limited to what can be obtained from CPET evaluations. Firstly, without specifically performing gait analysis, further biomechanical interpretations are limited, especially regarding the impact of balance, stability, and ground reaction forces. Since weight loss after bariatric surgery results in a reduction of waist and thigh circumferences as well as leg fat mass, gait analysis would allow more accurate considerations regarding how walking mechanics may affect the walking economy. Moreover, due to safety and feasibility reasons, all patients were recommended to hold the treadmill bar during all CPETs just to ensure balance, but they were instructed not to use the hold as a drag force. However, it is difficult to estimate the impact that holding the bar during treadmill walking could have had on the energy costs of walking since the absence of upper limb motion underestimates the real VO_2_ at a given stage. It is necessary to consider that the study sample also included very fragile patients with severe obesity for whom treadmill walking without the bar would simply not be feasible. Thus, in order to limit the bias among study participants, all patients underwent the same CPET procedure. Moreover, since all tests were performed by holding the bar during exercise in the same position and with the same timing, the consistency of measurements between pre- and post-SG could be guaranteed, and therefore the bias should be further limited.

The lack of data regarding body composition is a limiting factor in our study as it is not possible to analyze how much bariatric surgery has resulted in a loss of lean mass, affecting the walking economy. The experience in our center revealed that the determination of body composition with available methods is less reliable and technically more difficult in patients with severe obesity when compared to normal weight and mild obesity. Moreover, while bioelectrical impedance analysis and plicometry are limited in providing valid and/or reliable data on body composition, dual-energy X-ray absorptiometry and magnetic resonance imaging are technically not feasible in many patients with severe obesity who are candidates for bariatric surgery. Despite this, future experimental trials may specifically investigate whether walking economy is also associated with changes in fat/lean mass after SG. Finally, our data did not allow us to precisely identify the pathophysiological mechanism underlying the changes in the energy cost of walking, only suggesting that weight loss led to adaptations in the biomechanics of walking, making it more efficient and feasible in daily routine. Thus, future studies should provide a standardized long-term follow-up post-surgery with additional pathophysiological assessments for a larger sample size to specifically evaluate modifications and mechanisms of alterations in the walking economy after more sustained and stable weight loss.

## 5. Conclusions

SG significantly reduces subjects’ weight at six months follow-up, improving cardiovascular function, exercise capacity, and cardiorespiratory fitness. After SG, there is a reduction in the net energy cost of walking at different speeds and gradients in both genders and all obesity classes. SG may thus also have an important impact on long-term adherence to regular PA since it facilitates engagement in exercise training and NEAT. The walking economy assessment during functional evaluation adds value to the understanding of the global effect of SG for clinicians. Future studies, including gait analysis and body composition assessments before and after SG, are needed.

## Figures and Tables

**Table 1:** Anthropometric characteristics of the study participants pre- and post-sleeve gastrectomy.

	Age (years)	Height (cm)	Weight (kg)		BMI (kg/m^2^)		Waist Circumference (cm)	
			Pre	Post	Pre	Post	Pre	Post
All	44.1 ± 10.7	166.2 ± 9.5	117.9 ± 19.6	87.4 ± 15.8 *	42.5 ± 4.7	31.6 ± 4.2 *	127.8 ± 12.8	103.4 ± 13.4 *
All women	44.1 ± 10.6	162.7 ± 6.3	112.0 ± 14.6	83.1 ± 12.1 *	42.3 ± 4.4	31.4 ± 3.9 *	124.2 ± 11.0	100.1 ± 9.7 *
All men	44.0 ± 11.3	179.0 ± 8.1	139.3 ± 20.8	103.1 ± 17.6 *	43.5 ± 5.8	32.3 ± 5.1 *	138.8 ± 11.6	113.6 ± 17.7 *
Women BMI 35–40	45.8 ± 8.7	162.8 ± 5.3	100.2 ± 7.2	74.6 ± 7.9 *	37.8 ± 1.53	28.2 ± 2.72 *	115.4 ± 9.4	93.0 ± 7.5 *
Women BMI ≥ 40	43.3 ± 11.4	162.6 ± 6.8	117.8 ± 13.8	87.3 ± 11.7 *	44.5 ± 3.5	33.0 ± 3.4 *	128.1 ± 9.4	103.1 ± 9.0 *
Men BMI 35–40	42.0 ± 14.3	182 ± 7.1	124.3 ± 11.2	91.5 ± 14.4 *	37.4 ± 1.7	27.6 ± 3.5 *	127.5 ± 7.9	102.6 ± 15.0 *
Men BMI ≥ 40	45.0 ± 9.7	177.4 ± 8.3	146.8 ± 20.6	108.8 ± 16.4 *	46.5 ± 4.5	34.7 ± 4.0 *	144.8 ± 8.0	119.5 ± 16.4 *

BMI = body mass index. * *p* < 0.05 between pre- and post-SG evaluation.

**Table 2:** CPET parameters of the study participants pre- and post-sleeve gastrectomy.

	HR Rest (bpm)		HR Max (% Predicted)		METs Max		RER Max		VO_2_ Rest (L/min)		VO_2_ Rest (mL/kg/min)		VO_2_ Peak (L/min)		VO_2_ Peak (mL/kg/min)	
	Pre	Post	Pre	Post	Pre	Post	Pre	Post	Pre	Post	Pre	Post	Pre	Post	Pre	Post
All	81.6 ± 12.8	64.5 ± 9.5 *	91.1 ± 7.4	90.7 ± 7.4	1.13 ± 0.09	1.19 ± 0.10 *	1.13 ± 0.09	1.19 ± 0.10 *	0.42 ± 0.13	0.32 ± 0.11 *	3.6 ± 0.9	3.7 ± 1.2	2.46 ± 0.58	2.15 ± 0.55 *	20.8 ± 3.2	24.7 ± 5.2 *
All women	81.8 ± 12.6	65.0 ± 9.1 *	91.1 ± 7.5	90.5 ± 7.1	1.13 ± 0.10	1.17 ± 0.10 *	1.13 ± 0.10	1.17 ± 0.10 *	0.39 ± 0.11	0.30 ± 0.09 *	3.5 ± 0.9	3.6 ± 1.1	2.24 ± 0.35	1.96 ± 0.36 *	20.0 ± 2.5	23.8 ± 4.5 *
All men	80.7 ± 13.6	62.3 ± 10.9 *	90.7 ± 7.1	91.4 ± 8.5	1.14 ± 0.07	1.23 ± 0.10 *	1.14 ± 0.07	1.23 ± 0.10 *	0.54 ± 0.15	0.40 ± 0.13 *	3.9 ± 1.1	3.9 ± 1.3	3.26 ± 0.55	2.84 ± 0.59 *	23.6 ± 4.0	27.7 ± 6.2 *
Women BMI 35–40	80.6 ± 12.8	65.5 ± 9.0 *	90.9 ± 8.9	90.8 ± 8.8	1.14 ± 0.10	1.17 ± 0.11	1.14 ± 0.10	1.17 ± 0.11	0.34 ± 0.09	0.29 ± 0.08 *	3.4 ± 0.8	3.9 ± 1.1	2.08 ± 0.28	1.79 ± 0.30 *	20.8 ± 2.5	24.2 ± 4.8 *
Women BMI ≥ 40	82.4 ± 12.5	64.8 ± 9.2 *	91.3 ± 6.8	90.4 ± 6.2	1.12 ± 0.09	1.18 ± 0.10 *	1.12 ± 0.09	1.18 ± 0.10*	0.41 ± 0.11	0.30 ± 0.09 *	3.5 ± 0.9	3.5 ± 1.1	2.31 ± 0.36	2.05 ± 0.36 *	19.6 ± 2.4	23.7 ± 4.4 *
Men BMI 35–40	76.9 ± 18.5	59.0 ± 12.4 *	89.9 ± 6.4	87.5 ± 9.3	1.13 ± 0.08	1.21 ± 0.13	1.13 ± 0.08	1.21 ± 0.13	0.56 ± 0.11	0.36 ± 0.14 *	4.5 ± 1.2	3.9 ± 1.6	3.30 ± 0.64	2.85 ± 0.75 *	26.6 ± 5.1	30.7 ± 8.6
Men BMI ≥ 40	82.6 ± 10.4	64.0 ± 10.0 *	91.1 ± 7.5	93.4 ± 7.5 *	1.14 ± 0.06	1.25 ± 0.09 *	1.14 ± 0.06	1.25 ± 0.09 *	0.53 ± 0.15	0.42 ± 0.12 *	3.6 ± 0.8	3.9 ± 1.1	3.24 ± 0.51	2.84 ± 0.51 *	22.1 ± 2.2	26.2 ± 3.9 *

CPET = cardiopulmonary exercise test; BMI = body mass index; HR = heart rate; MET = metabolic equivalent of task; RER = respiratory exchange ratio; VO_2_ = oxygen uptake. * *p* < 0.05 between pre- and post-SG evaluation.

**Table 3:** CPET and net energy cost parameters of the study participants pre- and post-sleeve gastrectomy during the three protocol stages were also grouped by gender and obesity classes.

	CPET Parameters								Net Energy Cost of Walking			
	HR (bpm)		RER		VO_2_ (L/min)		VO_2_ (mL/kg/min)		Absolute (J/m)		Relative (J/kg/m)	
	Pre	Post	Pre	Post	Pre	Post	Pre	Post	Pre	Post	Pre	Post
Stage 0												
All	113.3 ± 14.0	89.8 ± 12.2 *	0.81 ± 0.06	0.80 ± 0.07 *	1.25 ± 0.29	0.81 ± 0.19 *	10.6 ± 1.8	9.3 ± 1.6 *	370.21 ± 114.19	218.82 ± 81.78 *	3.15 ± 0.86	2.50 ± 0.81 *
Women BMI 35–40	111.4 ± 13.6	90.5 ± 12.2 *	0.82 ± 0.06	0.79 ± 0.07	1.04 ± 0.19	0.70 ± 0.15 *	10.4 ± 2.1	9.5 ± 2.1 *	312.49 ± 84.71	183.73 ± 75.73 *	3.15 ± 0.94	2.48 ± 1.05 *
Women BMI ≥ 40	115.9 ± 13.7	91.2 ± 12.0 *	0.81 ± 0.05	0.79 ± 0.07 *	1.24 ± 0.24	0.80 ± 0.18 *	10.5 ± 1.7	9.2 ± 1.4 *	369.49 ± 109.69	222.04 ± 82.10 *	3.13 ± 0.86	2.51 ± 0.73 *
Men BMI 35–40	105.9 ± 20.1	82.9 ± 12.6 *	0.83 ± 0.13	0.83 ± 0.09	1.44 ± 0.25	0.92 ± 0.13 *	11.6 ± 1.7	10.1 ± 1.0 *	392.30 ± 137.75	252.79 ± 66.94 *	3.16 ± 1.07	2.77 ± 0.66 *
Men BMI ≥ 40	111.0 ± 10.3	86.7 ± 11.5 *	0.81 ± 0.05	0.80 ± 0.06	1.57 ± 0.28	0.99 ± 0.16 *	10.8 ± 1.6	9.1 ± 1.0 *	465.65 ± 77.74	253.24 ± 78.21 *	3.19 ± 0.67	2.33 ± 0.62 *
Stage 1/2												
All	119.2 ± 13.7	95.6 ± 12.6 *	0.85 ± 0.06	0.85 ± 0.07	1.40 ± 0.31	0.92 ± 0.21 *	11.9 ± 1.8	10.6 ± 1.5 *	437.32 ± 127.16	271.12 ± 90.18 *	3.73 ± 0.92	3.09 ± 0.82 *
Women BMI 35–40	118.1 ± 13.9	95.6 ± 12.3 *	0.86 ± 0.06	0.85 ± 0.07	1.18 ± 0.20	0.79 ± 0.13 *	11.8 ± 2.1	10.6 ± 1.9 *	377.91 ± 90.65	225.07 ± 73.03 *	3.80 ± 1.01	3.04 ± 1.01 *
Women BMI ≥ 40	121.3 ± 13.1	97.1 ± 12.6 *	0.85 ± 0.05	0.85 ± 0.06	1.36 ± 0.22	0.92 ± 0.20 *	11.7 ± 1.5	10.5 ± 1.5 *	424.33 ± 114.04	276.33 ± 90.14 *	3.63 ± 0.90	3.13 ± 0.77 *
Men BMI 35–40	111.5 ± 19.6	88.7 ± 11.8 *	0.87 ± 0.12	0.87 ± 0.09	1.61 ± 0.27	1.02 ± 0.15 *	13.0 ± 1.7	11.2 ± 0.9 *	475.39 ± 146.73	302.81 ± 86.61 *	3.83 ± 1.09	3.31 ± 0.81 *
Men BMI ≥ 40	117.5 ± 10.9	93.4 ± 13.2 *	0.86 ± 0.05	0.85 ± 0.06	1.80 ± 0.31	1.13 ± 0.19 *	12.3 ± 1.6	10.4 ± 0.9 *	571.96 ± 124.43	319.10 ± 88.45 *	3.92 ± 0.77	2.92 ± 0.60 *
Stage 1												
All	128.3 ± 13.8	104.8 ± 13.4 *	0.88 ± 0.06	0.88 ± 0.06	1.68 ± 0.45	1.12 ± 0.25 *	14.1 ± 1.9	12.7 ± 1.6 *	384.83 ± 126.92	241.07 ± 80.18 *	3.26 ± 0.77	2.75 ± 0.58 *
Women BMI 35–40	127.0 ± 13.6	104.7 ± 13.3 *	0.90 ± 0.08	0.87 ± 0.07	1.39 ± 0.22	0.94 ± 0.14 *	13.9 ± 2.3	12.7 ± 1.9 *	323.62 ± 68.01	201.27 ± 49.69 *	3.24 ± 0.74	2.71 ± 0.66 *
Women BMI ≥ 40	130.5 ± 13.1	106.5 ± 13.4 *	0.88 ± 0.06	0.87 ± 0.06	1.62 ± 0.26	1.1 ± 0.23 *	13.8 ± 1.7	12.6 ± 1.8 *	370.13 ± 79.70	244.04 ± 77.16 *	3.16 ± 0.60	2.77 ± 0.59 *
Men BMI 35–40	116.9 ± 21.1	95.6 ± 12.4 *	0.85 ± 0.06	0.89 ± 0.08	1.89 ± 0.34	1.19 ± 0.19 *	15.0 ± 1.8	13.0 ± 0.7 *	401.23 ± 122.01	256.50 ± 60.40 *	3.19 ± 0.80	2.79 ± 0.42 *
Men BMI ≥ 40	127.7 ± 10.4	103.4 ± 13.3 *	0.89 ± 0.05	0.88 ± 0.07	2.28 ± 0.68	1.38 ± 0.21 *	14.7 ± 1.6	12.7 ± 0.9 *	536.77 ± 208.60	294.15 ± 68.14 *	3.64 ± 1.21	2.70 ± 0.44 *

CPET = cardiopulmonary exercise test; BMI = body mass index; HR = heart rate; RER = respiratory exchange ratio; VO_2_ = oxygen uptake. * *p* < 0.05 between pre- and post-SG evaluation.

## Data Availability

The data that support the findings of this study are available from the corresponding author upon reasonable request.
